# The clinical effectiveness and safety of traditional Chinese medicine uremic clearance granule combined with high-flux hemodialysis in the treatment of uremic pruritus: A protocol for systematic review and meta analysis

**DOI:** 10.1097/MD.0000000000026423

**Published:** 2021-06-25

**Authors:** Ya-Fei Bai, Chun-Li Wang, Ming-Zhi Xu, Ming-Jiao Pan, Qing-Yi Sun, Ru-Man Chen

**Affiliations:** Blood Purification Center, Hainan General Hospital (Hainan Affiliated Hospital of Hainan Medical University), Xiuhua Road, Xiuying District, Haikou City, Hainan Province, P.R. China.

**Keywords:** high-flux hemodialysis, meta-analysis, safety, uremic clearance granule, uremic pruritus

## Abstract

**Background:**

Skin pruritus is a common complication in patients with uremia. When the hemodialysis time of patients is extended, and the probability of skin pruritus is greater. Patients often have the symptoms of skin pruritus intolerable, affecting the normal sleep and normal life of patients. The patients with uremic pruritus often constant scratching and pruritus skin, resulting in broken skin, and further symptoms such as infection, and subsequent skin shedding, prurigo nodularis, and other adverse complications, aggravating the patient's condition. Some patients will experience symptoms such as depression and insomnia due to skin pruritus, and simply scratching the skin lead to infection. Severely affected patients may even show suicidal tendency, endangering the physical and mental health of patients, and it is needed to give the effective treatment to patients. Hemodialysis is a common treatment for uremic pruritus, which can effectively relieve the pruritus symptoms of patients. The drugs can also relieve the symptoms and improve the degree of pruritus in patients. And some studies show that traditional Chinese medicine UCG combined with HFH in the treatment of uremic pruritus has a very good effect, Therefore, this study will systematically evaluate the clinical efficacy and safety of UCG combined with HFH and HFH alone in the treatment of uremic pruritus.

**Methods:**

Use computer to search English and Chinese databases, English databases include: PubMed, Web of Science, EMbase, The Cochrane Library. Chinese databases include: CNKI, CBM, WanFang Data and VIP databases, collecting the RCT on the clinical effectiveness and safety of UCG combined with HFH and HFH alone in the treatment of uremic pruritus. The retrieval time is from the beginning of each database to May 1, 2021. In order to improve the retrieval rate of the literature, the references cited in the included research are also collected and screened. Set Chinese and English as the search language. Two members of the research group independently collected, included and excluded the literatures. In case of disagreement, consulting the third party to assist in the judgment. For the literature with missing data, the original author should be contacted as far as possible to obtain complete data. Two evaluators evaluate the bias risk of included studies according to the Cochrane Handbook bias risk assessment tool for RCT. RevMan 5.3 software is used for statistical analysis and the forest plot is drawn to show the outcome indicators and funnel plot is drawn to show the publication bias.

**Results:**

This study evaluates the advantages and disadvantages of traditional Chinese medicine UCG combined with HFH and HFH alone in the treatment of uremic pruritus through the clinical effectiveness and safety-related indicators.

**Conclusion:**

This study will give a positive conclusion on the efficacy and safety of uremic clearance granule in the treatment of uremic pruritus, and the research results will be published in professional journals in the form of academic papers, thus benefiting more patients.

**Ethics and dissemination:**

This study belongs to meta-analysis and all data comes from academic papers published publicly in formal academic journals, so there are no ethical issues involved in this study and no ethical review or approval is required.

**OSF registration number:**

DOI 10.17605/OSF.IO/W8P5G.

## 1 Introduction

End-stage renal disease (ESRD) is commonly known as uremia, the imbalance of acid-base metabolism is more serious due to the loss of kidney function in patients.^[1,2]^ Hemodialysis is the preferred treatment for uremia. Hemodialysis can correct the body's metabolic disorders, remove toxins in the body, promote the balance of acid and alkali in the body, thereby improving the clinical symptoms. But the ordinary hemodialysis can only remove small molecular toxins in the blood, but it cannot remove the blood of the middle molecular and large molecular toxins. With the extension of dialysis time, and it is easy to lead to the accumulation of a variety of metabolic waste and toxins in the body, which leads to a series of complications.^[3,4]^ Uremic pruritus (UP) is one of the most common complications of ESRD. According to foreign literature, more than 40% of patients receiving peritoneal dialysis or hemodialysis have experienced skin pruritus of varying degrees and unbearable degrees.^[5]^ And domestic research data shows that the incidence of skin pruritus in patients with chronic renal failure in China is as high as 70%–90%, especially in patients entering hemodialysis period is higher.^[6]^ Uremic pruritus is a chronic, unbearable symptoms, often leading to insomnia and mood disorders. Patients can cause progressive damage to the skin due to excessive scratching, resulting in physical and mental discomfort, to reduce the life quality of patients, and even cause the increase of the hospitalization rate and long-term mortality rate.^[7]^ The incidence of UP is a complex pathophysiological process, and there is no specific pathogenesis, which may be related to immunity, inflammatory reaction, dry skin, histamine concentration and activity increase, peripheral neuropathy, ion disorders and so on.^[8]^

The treatment of ESRD should be carried out from the basic treatment and protection of kidney. The basic treatment includes diet treatment, hypertension control, water electrolyte and acid-base balance disorder, infection control, correction of renal bone disease, correction of renal anemia, prevention and treatment of cardiovascular complications and kidney replacement therapy, so as to further delay the development of the disease, protect kidney function, and improve the life quality. Different treatment strategies are made according to the disease stages and complications.^[9,10]^ At present, Western medicine has limited treatment for the disease, mainly through the optimization of dialysis program, skin hydration and lubrication, physiotherapy and antihistamine drugs, opioid receptor antagonists and other treatment measures. But the therapeutic effect is not obvious, and pruritus symptoms are difficult to obtain full control, and it may be accompanied by neurotoxicity, potential cancer risk and other side effects. Kidney transplantation is the most likely method to cure uremia skin itch, but it is facing a series of problems such as kidney scarcity, huge transplantation cost, easy infection and allogeneic rejection, so it is difficult to popularize the method in clinical. Traditional Chinese medicine has a unique advantage in this regard, Chinese medicine believes that UP is based on chronic kidney disease evolved. The disease lasts for a long time, leading to deficiency of spleen and kidney, and kidney deficiency, which eventually leads to the internal accumulation of dampness and toxin, endogenesis of damp and heat, inaccessibility outside and accumulation in skin, Therefore, it should use the traditional Chinese medicine of activating blood and relieving pruritus and dispersing wind and nourishing blood.^[11]^ Traditional Chinese medicine uremic clearance granule (UCG) is one of the first three types of pure Chinese medicine approved by the State Food And Drug Administration (SFDA) to treat kidney disease. The composition of the drug is as follows: plantain, mulberry white, ginger pinellia, rhubarb, atractylodes, polygonum multiflorum, astragalus, salvia miltiorrhiza, sophora flavescens, ligusticum wallichii, poria cocos, radix paeoniae alba. Among them, rhubarb has the function of clearing heat, promoting blood circulation and removing blood stasis; astragalus has anti-inflammatory and antioxidant effects; atractylodes has the effect of spleen, drying dampness and diuresis; mulberry white has anti-inflammatory, diuretic and detumescence effects; poria cocos has the effect of promoting urination; salvia miltiorrhiza has the effects of promoting blood circulation, removing blood stasis, dredging channels and relieving pain; ligusticum wallichii has the effect of promoting blood circulation and removing blood stasis.^[12]^ In recent years, studies have found that the effective active ingredients of UCG include astragaloside, emodin, salvianolic acid A, paeoniflorin, etc., and a variety of drugs work together to relieve clinical symptoms, reduce the level of inflammatory factors, anti-oxidative stress, and is also has a certain effect on renal anemia, calcium and phosphorus metabolism disorders and other complications.^[13–15]^

High-flux hemodialysis treatment has obvious dialysis effects on patients and it can reduce the incidence of UP. This is because the high-flux hemodialysis method is a new type of hemodialysis treatment that is further upgraded and optimized on the basis of the conventional hemodialysis treatment mode. Comparing to ordinary hemodialysis, high-flux hemodialysis uses a polymer membrane, and the biocompatibility is better, and it is conducive to avoiding blood in contact with dialysis membrane and produce inflammatory reactions, improve micro-inflammatory state, and its ultrafiltration coefficient is higher > 20 ml/(h·mmHg). The adsorption and water permeability are better, and the aperture of dialysis membrane is larger. It not only can remove inorganic phosphorus, SCr, BUN and other small molecular toxins in the body, but also can effectively remove the β2-MG and other medium and large molecular toxins, thereby alleviating pruritus symptoms.^[16]^ At present, uremic clearance granule and high-flux hemodialysis have been widely used by clinicians to treat UP, and some studies show that traditional Chinese medicine uremic clearance granule combined with high-flux hemodialysis in the treatment of uremic pruritus has a very good effect, Therefore, this study will systematically evaluate the clinical efficacy and safety of UCG combined with HFH and HFH alone in the treatment of uremic pruritus.

## 2 Methods

### 2.1 Protocol registration

The study has been registered on Open Science Framework. OSF registration number is DOI 10.17605/OSF.IO/W8P5G (https://osf.io/w8p5 g).

### 2.2 Inclusion and exclusion criteria

#### 2.2.1 Types of studies

Collecting randomized controlled studies on the effectiveness and safety of traditional Chinese medicine uremic clearance granule combined with high-flux hemodialysis and high-flux hemodialysis alone in the treatment of UP, regardless of whether the study is blinded or not, the language range includes Chinese and English.

#### 2.2.2 Types of participants

The study is conducted on all patients with uremia who met the inclusion and exclusion criteria while undergoing regular hemodialysis and developed symptoms of skin pruritus, with no restriction on age, gender or course of illness.

#### 2.2.3 Inclusion criteria

1. Maintenance hemodialysis patients who meet the diagnostic criteria of Western medicine and are diagnosed with UP.
2. Meet the diagnostic criteria of traditional Chinese medicine syndrome differentiation for chronic renal failure.
3. The treatment time of maintenance hemodialysis ≥ 12 months.
4. Those who have not undergone kidney transplantation, and have no severe primary diseases such as lung, liver, gallbladder, brain, cardiovascular, hematopoietic system, and endocrine and wasting diseases such as diabetes, malignant tumors, and tuberculosis.
5. No hormones or antihistamines are used in the 4 weeks before the study.

#### 2.2.4 Exclusion criteria

1. Case reports, conference papers, non-randomized controlled trials, animal experiments.
2. Combined with primary skin diseases, such as allergic dermatitis, eczema, urticaria, drug-induced rash, psoriasis, etc.
3. Combined with other systemic diseases, such as systemic lupus erythematosus, cholestasis, etc.
4. Tumor-related nephropathy, such as multiple myeloma.
5. Those who are allergic to the drugs used in this program.
6. People with mental illness or communication difficulties.

#### 2.2.5 Experimental interventions and control interventions

Experimental group: The traditional Chinese medicine uremic clearance granule combined with high-flux hemodialysis are used to treat UP.

Control group: High-flux hemodialysis is used to treat UP.

All patients are assisted with basic treatment, including diet treatment, control of hypertension, correction of water-electrolyte and acid-base balance disorders, control of infection, correction of renal bone disease, correction of renal anemia, prevention and treatment of cardiovascular complications, etc. There are no restrictions on the dosage, specifications and duration of the drug.

### 2.3 Types of outcome measures

#### 2.3.1 Primary outcomes

1. The skin pruritus degree scoring system, the scoring standards are as follows: score once a week, divided into the morning (wake up in the morning to noon) and afternoon (noon to before bedtime).^[17]^
  Pruritus degree scoring: ① Scratching is not required for skin pruritus (1 point). ② Scratching is required without scratches or scratches (2 points). ③ Itching persists without relief after scratching (3 points). ④ Symptoms of skin pruritus persist after scratching (4 points). ⑤ People who are irritable (5 points).
  Distribution range scoring: ① single part (1 point). ② scattered multiple parts (2 points). ③ general pruritus (3 points).
  Attack frequency scoring: 4 episodes of pruritus symptoms in a short period of time (each time ≤10 min) or 1 prolonged episode (a wake-up from itching during sleep is 2 points, the highest is 14 points. The highest possible total score for 24 h a day is 40 points (degree + part + frequency sum).
2. Pittsburgh sleep quality index (PSQI): There have been literature reports that there is a significant negative correlation between sleep quality and pruritus degree, and PSQI scale is used for sleep quality evaluation in patients.
3. Kidney disease quality of life short form (KDQOL-SF^tm^):
  The KDQOL-SF^tm^ 1.3 scale is used to investigate the life quality of patients.^[18]^
4. Clinical effectiveness: Healed: skin pruritus symptoms and signs are completely improved, and the pruritus scoring is reduced by 95% or more;
  Significantly effective: skin pruritus symptoms and signs are significantly improved, and the pruritus scoring is reduced by 70% or more;
  Effective: skin pruritus symptoms and signs have been improved, and the pruritus scoring is reduced by 30% or more;
  Ineffective: skin pruritus symptoms and signs are not improved, and the pruritus scoring is reduced by less than 30%.

#### 2.3.2 Additional outcomes

White blood cell (WBC), hemoglobin (Hb), serum creatinine (SCr), blood urea nitrogen (BUN), parathyroid hormone (PTH), beta-2-microglobulin (β2-MG), C-reactive protein (CRP), interleukin- 6 (IL-6), tumor necrosis factor-α (TNF-α) and adverse reaction rate.

### 2.4 Search strategy

Use computer to search English and Chinese databases, English databases include: PubMed, Web of Science, EMbase, The Cochrane Library. Chinese databases include: CNKI, CBM, WanFang Data and VIP databases, collecting the RCT on the clinical effectiveness and safety of UCG combined with HFH and HFH alone in the treatment of uremic pruritus. The retrieval time is from the beginning of each database to May 1, 2021. In order to improve the retrieval rate of the literature, the references cited in the included research are also collected and screened. Set Chinese and English as the search language. The complete retrieval strategy is presented by searching the literature using the Cochrane Library, and the results are shown in the Table 1.

**Table 1:** The results are obtained by searching the Cochrane Library.

Database	Number	Search items
The Cochrane Library	1	(Uremic clearance granule) OR (Niaoduqing granule): Title, Abstract, Keyword
	2	(High-flux hemodialysis) OR (Renal Dialysis) OR (Dialyses, Renal) OR (Renal Dialyses) OR (Dialysis, Renal) OR (Hemodialysis) OR (Hemodialyses) OR (Dialysis, Extracorporeal) OR (Dialyses, Extracorporeal) OR (Extracorporeal Dialyses) OR (Extracorporeal Dialysis): Title, Abstract, Keyword
	3	(Uremic pruritus) OR (Uremias pruritis) OR (Itching): Title, Abstract, Keyword
	4	1 AND 2 AND 3

Table 1. The results of search strategy in the Cochrane Library.

### 2.5 Data collection and analysis

**Figure 1:**
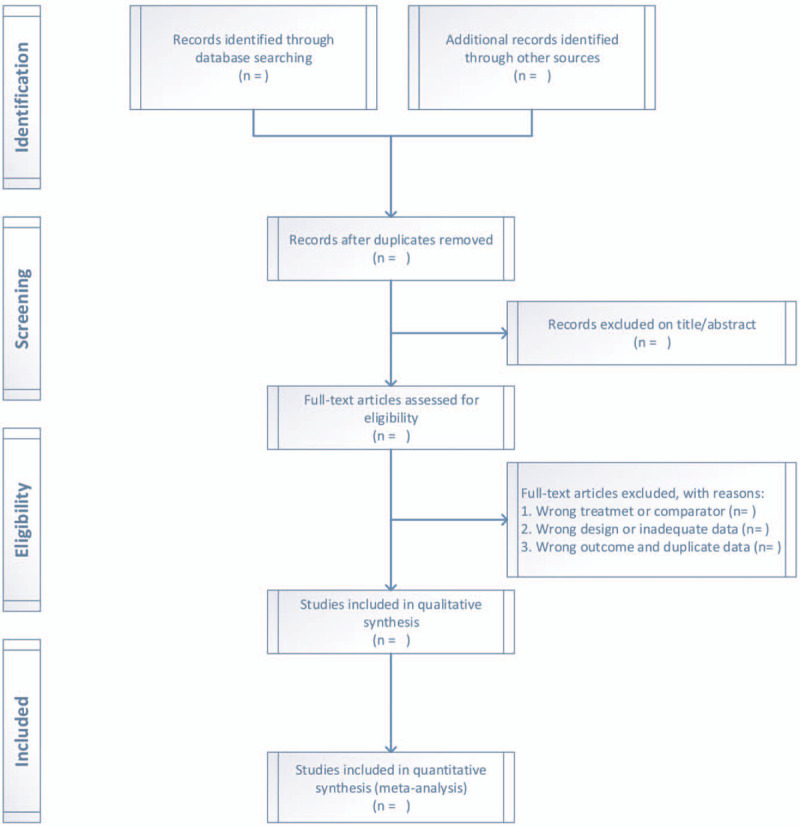
The results of the process of collecting and collating relevant literatures.

According to the above criteria, two members of the research group independently collected, included and excluded the literatures, and then reading the full text of the literature that may meet the requirements, then excluding the literature again, such as the inconsistent research type, the inconsistent control and intervention measures, and the incomplete experimental data, and the remaining literature is included in the study. Cross-checking the search, screening, and integration processes, and if there are differences, asking the third researcher to join the discussion and decide it. The extracted information of the selected literatures includes: the first author of the literature, the number of people in the experimental/control group, the age of the experimental /control group, the intervention content of the experimental group, the control measures, the time of the intervention, the outcome indicators, etc. Figure 1 shows the results of the process of collecting and collating relevant literatures.

### 2.6 Risk of bias assessment

Using the improved Jadad scale, the methodological quality evaluation of the included studies is carried out, and the contents of the evaluation include: random method, allocation hiding, blind method and missed visit. The highest score of Jadad evaluation is 7 points, of which 4 points and above are regarded as high-quality study, and 3 points and below are regarded as low-quality study.

1. Random: Determining whether the study use a random method, if so, to further determine whether the random method used in the study is correct, the use of random and the method is correct as a score, the use of random but the researcher did not elaborate on the random method as a score, did not use a random or random method incorrect as a score.
2. Allocation hidden: The judgment of randomized allocation hidden includes whether the allocation hidden method is used, if so, further determine whether the allocation hidden method is correct, the allocation hidden is used and the method is correct as a score, the allocation hidden is used but the researcher did not elaborate as a score, allocation hiding is not used or the allocation hiding method incorrect as a score.
3. Blind method: The judgment of blind method includes whether the blind method is used, if so, to further judge whether the use of blind method is correct, the blind method is used and the method is correct as a score, the blind method is used but the researcher did not elaborate as a score, the blind method is not used or blind method is not correct as a score.
4. Withdrawal and missed visit: If a patient withdraws or misses visit an interview during the study, the reasons for the withdrawal or missed visit shall be specified in detail, as well as the number of patients who have withdrawn or missed visit, the correct record as a score, no record or no explanation as a score. If there is an withdraw or misses visit in the study, the researcher should use intention-to-treat (ITT).

### 2.7 Statistical analysis

Revman5.3 software was used for statistical analysis of the test results. The binary variables were analyzed by relative risk (RR), and the continuous variables were analyzed by mean difference (MD) or standard mean difference (SMD), Both were expressed by 95% confidence interval (CI). If *P* > 0.1 and I^2^ < 50%, the results are considered to be homogeneous, and the fixed effect model (FEM) is used for combined analysis; If *P* ≤ 0.1 and I^2^ ≥ 50%, it means that there is heterogeneity among the research results. Try to find out the factors that cause the heterogeneity, and use subgroup analysis to calculate the statistics with heterogeneity. If there are no factors that cause clinical or methodological differences, or after sub-group analysis and processing, the results of the same type are still heterogeneous, use a random-effect model (REM). Combined statistics need to use the method of hypothesis test for Z test, *P* < .05 can be considered that the outcome index is statistically significant. The results of meta-analysis were shown by forest map, in which the vertical line of forest map was invalid, and its value was not statistically significant. Each horizontal line is the upper and lower limit of 95% confidence interval of each study, and its length represents the size of the confidence interval, in which the square represents the statistics of each test, and the square size represents the weight of the test. When the horizontal line falls on the left or right side of the vertical line, it indicates that the test has statistical significance; On the contrary, there was no statistical significance. We will contact the original author of the paper to obtain the complete data if there is definite data in the included literature.

### 2.8 Sensitivity analysis

Sensitivity analysis is required to assess the stability of meta-analysis results. This paper mainly excludes any study one by one, and then combines the analysis of a result indicator to determine the stability of the meta-analysis results of the indicator. If the meta-analysis results are not very different after excluding a study, the range of 95% CI does not contain “0” or “1”, which is equivalent to the combined effect amount *P* < .05, that is, the combined effect results are statistically significant, indicating that the meta-analysis results of the indicator are stable. On the other hand, it is unstable. When the conclusion is contrary, the rejected literature should be analyzed in detail.

### 2.9 Subgroup analysis

If there is a large heterogeneity in the present outcome indicators, we will conduct subgroup analysis in the following aspects, such as drug dosage, duration of disease, duration of maintenance hemodialysis. But the premise is that all data can be obtained or calculated from the selected literature.

### 2.10 Publication bias

A funnel chart is used to evaluate publication bias. The chart uses the standard error (SE) of the effect size as the abscissa, and the effect size as the ordinate. The combined effect size of each study is used as the central axis and the X axis is a horizontal line. Draw a scatter plot based on the corresponding data of each study, and roughly judge whether the selected documents have publication bias and the size of the publication bias according to the left-right symmetry of the funnel chart and the degree of concentration to the midline. If the number and distribution of the scattered points on both sides are basically the same, it means that there is no publication bias; on the contrary, there is publication bias.

### 2.11 Ethics and dissemination

This study belongs to meta-analysis and all data comes from academic papers published publicly in formal academic journals, so there are no ethical issues involved in this study and no ethical review or approval is required.

### 2.12 Confidence in cumulative evidence

GRADE classification uses five degrading factors including research limitation, inconsistency, indirectness, imprecision, and publication bias to grade the quality of evidence for outcome indicators, which are divided into four levels: high, intermediate, low, and very low, among which randomized control The RCT evidence quality rating is present to be high, with one level down to intermediate, two level down to low, and three level down to very low. The GRADE grading is completed independently by two researchers, and cross-checked after completion. In case of disagreement, the third researcher will arbitrate.

## 3 Discussion

The progression of chronic renal failure to the end-stage is called uremia, which can lead to a series of abnormal function changes in the body, especially the damage to the cardiovascular system, respiratory system, gastrointestinal system, blood system, bone system, and the threat to the life and health of patients is increasing. For this type of end-stage renal disease patients, there is no special drug treatment, so maintenance hemodialysis is a more commonly used treatment. Through the principle of dispersion, ultrafiltration, adsorption and convection, the patient's blood and dialysis fluid can be exchanged, which can remove metabolic waste in the body to maintain the electrolyte and acid-base balance, and thus alleviate the symptoms of renal dysfunction. However, conventional hemodialysis treatment can only remove some small molecules of toxins, but it has weak or almost no effect on medium-sized molecules and macromolecular protein binding toxins. Thus, the effect of conventional hemodialysis treatment is general, and long-term hemodialysis treatment on patients’ compliance and tolerance caused adverse effects. Therefore, for the treatment of uremia patients, it should be upgraded and optimized based on routine dialysis treatment to strengthen the elimination of toxins in the body.

UP is one of the common complications in patients with maintenance hemodialysis, with the incidence rate of up to 60% to 90%.^[19]^ The serious skin pruritus affects the daily life and sleep quality of patients, and some UP patients may experience anxiety, depression, and even suicidal tendencies, which is an important factor in the increase of death rate in dialysis patients. The incidence of UP is a complex pathophysiological process, there is no specific pathogenesis, may be related to the following conditions:^[20,21]^

1. immune and inflammatory reactions have a lot of inflammatory substances in the serum of UP patients, including IL-6, tumor necrosis factor, C-reactive protein, CXC- chemokine receptor 3, interferon-γ and so on. The levels of interferon and other factors in patients with pruritus are higher than those in patients without uremia.
2. uremic toxin accumulation: the common electrolyte disorder in patients with end-stage renal disease is low calcium and high phosphorus. The level of calcium and phosphorus rises and settles in the skin, which stimulates the peripheral nerves of the skin and causes pruritus.
3. histamine level: histamine has strong activity, which can expand skin and capillaries, increase vascular permeability, contract smooth muscle and increase gland secretion activity; and histamine can stimulate nerve endings, pain and pruritus symptoms. Under normal circumstances, histamine can be excreted through the kidney, while histamine accumulates in patients with end-stage renal failure; the plasma histamine in normal body is rapidly inactivated in microvascular endothelial cells. Uremic patients have inactivation disorder due to microvascular injury, which further increases the concentration of histamine and causes pruritus.
4. secondary parathyroid hyperthyroidism: disorders of calcium and phosphorus metabolism can easily promote the excessive secretion of parathyroid hormone. A large amount of parathyroid hormone stimulates mast cells, increasing the release of histamine and causing itching. Opioid disorders: opioids can induce the release of histamine from basophils and cause itching; the use of opioid antagonists can alleviate the symptoms of pruritus in patients.

Therefore, treatments have been the key research direction for the treatment of UP based on the above mechanisms.

Traditional Chinese medicine UCG and HFH treat UP has been applied to the treatment of UP and achieved good results. So far, domestic scholars have isolated and extracted a variety of effective bioactive ingredients from uremic clearance granule, including astragaloside, isoflavone, emodin, paeoniflorin and salvianolic acid A and so on. Modern research shows that uremic clearance granule has the effects that reduce SCr, BUN, improve plasma albumin, improve renal anemia, calcium and phosphorus, etc., effectively delay the progress of chronic renal failure, greatly improve the life quality of patients. In addition, it can enhance the phagocytosis of macrophages, promote the transformation of lymphocytes, induce cells to produce interferon, improve non-specific immune function.^[22,23]^ Comparing with ordinary hemodialysis treatment, high-flux hemodialysis mode uses high-flux filters with high-solute diffusion properties and permeability synthetic membranes, and the ultrafiltration coefficient and filter membrane area are significantly improved relative to ordinary filters, which can be used to deliver dialysis to dialysis fluids with larger relative molecular mass. High-flux filter for relative molecular mass of large and medium-sized molecular substances have a good removal effect, and the perfusion device is also equipped with a solid adsorbent, which has a strong non-specific adsorption effect. The substance to be removed has a good adsorption effect, and good removal effect on internal and external toxins, thereby significantly improving the pruritus symptoms of patients with uremia.^[24]^

At present, there is a dispute about the clinical effectiveness and safety of traditional Chinese medicine uremic clearance granule in the treatment of UP. Therefore, this study used the method of meta-analysis to evaluate the clinical effectiveness and safety of traditional Chinese medicine UCG combined with HFH and HFH alone in the treatment of uremic pruritus, and to provide clinicians with evidence-based evidence to help clinicians choose the right UP treatment method.

## Author contributions

**Conceptualization:** Ya-Fei Bai, Ru-Man Chen.

**Data curation:** Ya-Fei Bai, Chun-Li Wang, Ming-Zhi Xu, Ming-Jiao Pan.

**Formal analysis:** Ya-Fei Bai, Chun-Li Wang, Ming-Zhi Xu.

**Funding acquisition:** Ru-Man Chen.

**Project administration:** Ru-Man Chen.

**Resources:** Ya-Fei Bai, Chun-Li Wang, Ming-Zhi Xu, Ming-Jiao Pan.

**Software:** Ming-Zhi Xu, Ming-Jiao Pan, Qing-Yi Sun.

**Supervision:** Ya-Fei Bai.

**Writing – original draft:** Ya-Fei Bai, Chun-Li Wang, Ming-Zhi Xu, Ming-Jiao Pan, Qing-Yi Sun.

**Writing – review & editing:** Ru-Man Chen.
